# Supplementary material for: Clinically Isolated β-Lactam-Resistant Gram-Negative Bacilli in a Philippine Tertiary Care Hospital Harbor Multi-Class β-Lactamase Genes
Source: Pathogens. 2023 Aug 8;12(8):1019. doi: 10.3390/pathogens12081019 (PMC10459468; doi:10.3390/pathogens12081019)
Supplement: Supplementary file 1 [file pathogens-12-01019-s001.zip › pathogens-2519522-supplementary.pdf]

Clinically isolated β–lactam–resistant Gram–negative bacilli in a Philippine tertiary care hospital harbor multi–class β–lactamase genes

Alecks Megxel S. Abordo,[a,†] Mark B. Carascal,[a,†] Roland Remenyi,[a] Doralyn S. Dalisay[b,d], Jonel P. Saludes[c,d]
[a]Clinical and Translational Research Institute, The Medical City, Ortigas Avenue, 1605 Pasig City, Philippines
[b]Center for Chemical Biology and Biotechnology (M3M2) and Department of Biology, University of San Agustin, Gen. Luna Street, 5000 Iloilo City, Philippines
[c]Center for Natural Drug Discovery and Development (CND3) and Department of Chemistry, University of San Agustin, Gen. Luna Street, 5000 Iloilo City, Philippines
[d]Balik Scientist Program, Philippine Council for Health Research and Development, Department of Science and Technology, 1631 Taguig City, Philippines
[†]These authors contributed equally to the work

Corresponding Author: Jonel P. Saludes, Center for Natural Drug Discovery and Development (CND3) and Department of Chemistry, University of San Agustin, Gen. Luna Street, 5000 Iloilo City, Philippines
Email address: jsaludes@usa.edu.ph

Supplementary Data

Table S1. Primers, PCR conditions, positive controls, and reference literature used for the amplification of the 16S rRNA and target β–lactamase genes.

Gene Targets	Primer Sequence (5'–3')<br>F– Forward, R– Reverse	Amplicon Size (bp)	Annealing Temperature (°C)	Positive Control	Primer Reference
16S rRNA	F– AGAGTTTGATCCTGGCTCAG<br>R– GGTTACCTTGTTACGACTT	1350	58	*S. aureus* ATCC 29213	[29]
bla_{TEM}	F–CATTTCCGTGTCGCCCTTATTC<br>R– CGTTCATCCATAGTTGCCTGAC	800	61	*E. coli* ATCC 35218	[26]
bla_{SHV}	F– TTATCTCCCTGTTAGCCACC<br>R– GATTTGCTGATTTCGCTCGG	797	51	*K. pneumoniae* ATCC 700603	[30]
$bla_{CTX–M}$	F– TTAGGAAGTGTGCCGCTGTA<br>R– CGATATCGTTGGTGGTGCCAT	688	60	*E. coli* NCTC 13353	[26]
bla_{FOX}	F– AACATGGGGTATCAGGGAGATG<br>R– CAAAGCGCGTAACCGGATTGG	190	60	*K. pneumoniae* ATCC 2146	[31]
bla_{DHA}	F– TGATGGCACAGCAGGATATTC<br>R– GCTTTGACTCTTTCGGTATTCG	997	60	*E. coli* TMC EF1	[26]
bla_{IMP}	F–GGAATAGAGTGGCTTAATTCTC<br>R– GGTTTAACAAAACAACCACC	233	52	*E. coli* NCTC 13476	[27]
bla_{VIM}	F– ATGTTAAAAGTTATTAGTAGTTTATTG<br>R– CTACTCGGCGACTGAGC	801	57	*K. pneumoniae* NCTC 13440	[32]
bla_{KPC}	F– ATGTCACTGTATCGCCGTCT<br>R– TTACTGCCCGTTGACGCCC	882	63	*K. pneumoniae* ATCC BAA 1705	[33]
bla_{NDM}	F–GGTTTGGCGATCTGGTTTTC<br>R– CGGAATGGCTCATCACGATC	621	60	*K. pneumoniae* ATCC BAA 2146	[32]
$bla_{OXA–1}$	F– TCTGTTGTTTGGGTTTCGC<br>R– TCTATGGTGTTTTCTATGGCTG	245	52	*E. coli* ATCC BAA 2469	[35]
$bla_{OXA–2}$	F– ATTTCAAGCCAAAGGCACGA<br>R– GCCACTCAACCCATCCTACC	569	52	*K. pneumoniae* ATCC 700603	[34]
$bla_{OXA–23–like}$	F– CTATTTTTGTCGTGTACAGAGC<br>R– GGATCACAACAACTAAAAGCACTG	1008	62	*A. baumannii* ATCC BAA 1605	[35]
$bla_{OXA–24–like}$	F– GGTTAGTTGGCC CCCTTAAA<br>R– AGTTGAGCGAAA AGGGGATT	249	53	*A. baumannii* ATCC BAA 1605	[28]
$bla_{OXA–48–like}$	F–TTGGTGGCATCGATTATCGG<br>R– GAGCACTTCTTTTGTGATGGC	744	52	*K. pneumoniae* NCTC 13442	[36]
$bla_{OXA–51–like}$	F– CCATGGCAATGAACATTAAAGCACTC TTAC<br>R– CTATAAAATACCTAATTGTTCT	825	51	*A. baumannii* ATCC BAA 1605	[37]

Gene Targets	Primer Sequence (5’–3’)<br>F– Forward, R– Reverse	Amplicon Size (bp)	Annealing Temperature (°C)	Positive Control	Primer Reference
bla$_{OXA-58-like}$	F– AAGTATTGGGGCTTGTGCTG<br>R– CCCCTCTGCGCTCTACATAC	599	58	*A. baumannii* TMC 32	[38]

Table S2. GenBank® Accession Numbers of the sequenced sample isolate genes. The genes presented include 16S rRNA– 16S ribosomal ribonucleic acid, CTX–M– Cefotaximase–Munich, TEM– Temoneira β–lactamase, SHV– Sulfhydryl reagent variable β–lactamase, NDM– New Delhi metallo–β–lactamase, IMP– Imipenemase, DHA– Dharhan β–lactamase, OXA– Oxacillinase.

Organism/ Detected Gene	GenBank Accession Number
Klebsiella pneumoniae KF1 16S rRNA	OQ117104
Klebsiella pneumoniae KF2 16S rRNA	OQ117105
Klebsiella pneumoniae KF3 16S rRNA	OQ117106
Klebsiella pneumoniae KF4 16S rRNA	OQ117107
Klebsiella pneumoniae KM1 16S rRNA	OQ117108
Klebsiella pneumoniae KM2 16S rRNA	OQ117109
Klebsiella pneumoniae KM3 16S rRNA	OQ117110
Klebsiella pneumoniae KM4 16S rRNA	OQ117111
Escherichia coli EF1 16S rRNA	OQ117096
Escherichia coli EF2 16S rRNA	OQ117097
Escherichia coli EF3 16S rRNA	OQ117098
Escherichia coli EF4 16S rRNA	OQ117099
Escherichia coli EM1 16S rRNA	OQ117100
Escherichia coli EM2 16S rRNA	OQ117101
Escherichia coli EM3 16S rRNA	OQ117102
Escherichia coli EM4 16S rRNA	OQ117103
Acinetobacter baumannii AF1 16S rRNA	OQ117089
Acinetobacter baumannii AF2 16S rRNA	OQ117090
Acinetobacter baumannii AF3 16S rRNA	OQ117091
Acinetobacter baumannii AF4 16S rRNA	OQ117092
Acinetobacter baumannii AM1 16S rRNA	OQ117093
Acinetobacter baumannii AM2 16S rRNA	OQ117094
Acinetobacter baumannii AM3 16S rRNA	OQ117095
Pseudomonas aeruginosa AF2 16S rRNA	OQ117112
Pseudomonas aeruginosa AF3 16S rRNA	OQ117113
Pseudomonas aeruginosa AF4 16S rRNA	OQ117114
Pseudomonas aeruginosa AM2 16S rRNA	OQ117115
Pseudomonas aeruginosa AM3 16S rRNA	OQ117116
Pseudomonas aeruginosa AM4 16S rRNA	OQ117117
Escherichia coli EF1 $bla_{CTX–M}$	OQ096344
Escherichia coli EF2 $bla_{CTX–M}$	OQ096345
Escherichia coli EF3 $bla_{CTX–M}$	OQ096346
Escherichia coli EF4 $bla_{CTX–M}$	OQ096347
Escherichia coli EM1 $bla_{CTX–M}$	OQ096340
Escherichia coli EM2 $bla_{CTX–M}$	OQ096341

Organism/ Detected Gene	GenBank Accession Number
Escherichia coli EM3 *bla*$_{CTX–M}$	OQ096342
Escherichia coli EM4 *bla*$_{CTX–M}$	OQ096343
Klebsiella pneumoniae KF1 *bla*$_{CTX–M}$	OQ096352
Klebsiella pneumoniae KF2 *bla*$_{CTX–M}$	OQ096353
Klebsiella pneumoniae KF3 *bla*$_{CTX–M}$	OQ096354
Klebsiella pneumoniae KF4 *bla*$_{CTX–M}$	OQ096355
Klebsiella pneumoniae KM1 *bla*$_{CTX–M}$	OQ096348
Klebsiella pneumoniae KM2 *bla*$_{CTX–M}$	OQ096349
Klebsiella pneumoniae KM3 *bla*$_{CTX–M}$	OQ096350
Klebsiella pneumoniae KM4 *bla*$_{CTX–M}$	OQ096351
Pseudomonas aeruginosa PM3 *bla*$_{CTX–M}$	OQ096356
Acinetobacter baumannii AM1 *bla*$_{CTX–M}$	OQ096357
Acinetobacter baumannii AM3 *bla*$_{CTX–M}$	OQ096358
Escherichia coli EF1 *bla*$_{DHA}$	OQ096359
Escherichia coli EF2 *bla*$_{DHA}$	OQ096360
Escherichia coli EF1 *bla*$_{IMP}$	OQ096361
Escherichia coli EF4 *bla*$_{IMP}$	OQ096362
Klebsiella pneumoniae KF4 *bla*$_{IMP}$	OQ096364
Klebsiella pneumoniae KM3 *bla*$_{IMP}$	OQ096363
Pseudomonas aeruginosa PF2 *bla*$_{IMP}$	OQ096365
Pseudomonas aeruginosa PF3 *bla*$_{IMP}$	OQ096366
Pseudomonas aeruginosa PF4 *bla*$_{IMP}$	OQ270746
Pseudomonas aeruginosa PM2 *bla*$_{IMP}$	OQ270743
Pseudomonas aeruginosa PM3 *bla*$_{IMP}$	OQ270744
Pseudomonas aeruginosa PM4 *bla*$_{IMP}$	OQ270745
Acinetobacter baumannii AF1 *bla*$_{IMP}$	OQ096368
Acinetobacter baumannii AF2 *bla*$_{IMP}$	OQ270742
Acinetobacter baumannii AF4 *bla*$_{IMP}$	OQ096369
Acinetobacter baumannii AM3 *bla*$_{IMP}$	OQ096367
Escherichia coli EF2 *bla*$_{NDM}$	OQ096374
Escherichia coli EF3 *bla*$_{NDM}$	OQ096375
Escherichia coli EF4 *bla*$_{NDM}$	OQ096376
Escherichia coli EM1 *bla*$_{NDM}$	OQ096370
Escherichia coli EM2 *bla*$_{NDM}$	OQ096371
Escherichia coli EM3 *bla*$_{NDM}$	OQ096372
Escherichia coli EM4 *bla*$_{NDM}$	OQ096373
Klebsiella pneumoniae KF2 *bla*$_{NDM}$	OQ096380
Klebsiella pneumoniae KF4 *bla*$_{NDM}$	OQ270747
Klebsiella pneumoniae KM2 *bla*$_{NDM}$	OQ096377
Klebsiella pneumoniae KM3 *bla*$_{NDM}$	OQ096378
Klebsiella pneumoniae KM4 *bla*$_{NDM}$	OQ096379

Organism/ Detected Gene	GenBank Accession Number
Pseudomonas aeruginosa PF2 *bla*$_{NDM}$	OQ096382
Pseudomonas aeruginosa PM3 *bla*$_{NDM}$	OQ096381
Acinetobacter baumannii AF1 *bla*$_{NDM}$	OQ096385
Acinetobacter baumannii AF2 *bla*$_{NDM}$	OQ096386
Acinetobacter baumannii AM1 *bla*$_{NDM}$	OQ096383
Acinetobacter baumannii AM3 *bla*$_{NDM}$	OQ096384
Escherichia coli EF1 *bla*$_{OXA–1}$	OQ096391
Escherichia coli EF2 *bla*$_{OXA–1}$	OQ096392
Escherichia coli EF3 *bla*$_{OXA–1}$	OQ096393
Escherichia coli EF4 *bla*$_{OXA–1}$	OQ096394
Escherichia coli EM1 *bla*$_{OXA–1}$	OQ096387
Escherichia coli EM2 *bla*$_{OXA–1}$	OQ096388
Escherichia coli EM3 *bla*$_{OXA–1}$	OQ096389
Escherichia coli EM4 *bla*$_{OXA–1}$	OQ096390
Klebsiella pneumoniae KF2 *bla*$_{OXA–1}$	OQ270748
Klebsiella pneumoniae KF3 *bla*$_{OXA–1}$	OQ270749
Klebsiella pneumoniae KF4 *bla*$_{OXA–1}$	OQ270750
Klebsiella pneumoniae KM1 *bla*$_{OXA–1}$	OQ096395
Klebsiella pneumoniae KM2 *bla*$_{OXA–1}$	OQ096396
Klebsiella pneumoniae KM3 *bla*$_{OXA–1}$	OQ096397
Klebsiella pneumoniae KM4 *bla*$_{OXA–1}$	OQ096398
P. aeruginosa PF2 *bla*$_{OXA–1}$	OQ270753
P. aeruginosa PF3 *bla*$_{OXA–1}$	OQ270754
P. aeruginosa PM3 *bla*$_{OXA–1}$	OQ270751
P. aeruginosa PM4 *bla*$_{OXA–1}$	OQ270752
Escherichia coli EF1 *bla*$_{OXA–24–like}$	OQ096438
Escherichia coli EF4 *bla*$_{OXA–24–like}$	OQ096439
Escherichia coli EM4 *bla*$_{OXA–24–like}$	OQ096437
Klebsiella pneumoniae KF4 *bla*$_{OXA–24–like}$	OQ096443
Klebsiella pneumoniae KM1 *bla*$_{OXA–24–like}$	OQ096440
Klebsiella pneumoniae KM3 *bla*$_{OXA–24–like}$	OQ096441
Klebsiella pneumoniae KM4 *bla*$_{OXA–24–like}$	OQ096442
Pseudomonas aeruginosa PF2 *bla*$_{OXA–24–like}$	OQ096447
Pseudomonas aeruginosa PM2 *bla*$_{OXA–24–like}$	OQ096444
Pseudomonas aeruginosa PM3 *bla*$_{OXA–24–like}$	OQ096445
Pseudomonas aeruginosa PM4 *bla*$_{OXA–24–like}$	OQ096446
Acinetobacter baumannii PF1 *bla*$_{OXA–24–like}$	OQ096451
Acinetobacter baumannii PF2 *bla*$_{OXA–24–like}$	OQ096452
Acinetobacter baumannii AF3 *bla*$_{OXA–24–like}$	OQ096453
Acinetobacter baumannii AF4 *bla*$_{OXA–24–like}$	OQ096454
Acinetobacter baumannii AM1 *bla*$_{OXA–24–like}$	OQ096448

Organism/ Detected Gene	GenBank Accession Number
Acinetobacter baumannii AM2 *bla*$_{OXA-24-like}$	OQ096449
Acinetobacter baumannii AM3 *bla*$_{OXA-24-like}$	OQ096450
Acinetobacter baumannii AF1 *bla*$_{OXA-51-like}$	OQ096402
Acinetobacter baumannii AF2 *bla*$_{OXA-51-like}$	OQ096403
Acinetobacter baumannii AF3 *bla*$_{OXA-51-like}$	OQ096404
Acinetobacter baumannii AF4 *bla*$_{OXA-51-like}$	OQ096405
Acinetobacter baumannii AM1 *bla*$_{OXA-51-like}$	OQ096399
Acinetobacter baumannii AM2 *bla*$_{OXA-51-like}$	OQ096400
Acinetobacter baumannii AM3 *bla*$_{OXA-51-like}$	OQ096401
Klebsiella pneumoniae KF1 *bla*$_{SHV}$	OQ096410
Klebsiella pneumoniae KF2 *bla*$_{SHV}$	OQ096411
Klebsiella pneumoniae KF4 *bla*$_{SHV}$	OQ096412
Klebsiella pneumoniae KM1 *bla*$_{SHV}$	OQ096406
Klebsiella pneumoniae KM2 *bla*$_{SHV}$	OQ096407
Klebsiella pneumoniae KM3 *bla*$_{SHV}$	OQ096408
Klebsiella pneumoniae KM4 *bla*$_{SHV}$	OQ096409
Pseudomonas aeruginosa PF3 *bla*$_{SHV}$	OQ096413
Acinetobacter baumannii AF2 *bla*$_{SHV}$	OQ096414
Acinetobacter baumannii AF3 *bla*$_{SHV}$	OQ096417
Acinetobacter baumannii AF4 *bla*$_{SHV}$	OQ096418
Acinetobacter baumannii AM1 *bla*$_{SHV}$	OQ096415
Acinetobacter baumannii AM3 *bla*$_{SHV}$	OQ096416
Escherichia coli EF1 *bla*$_{TEM}$	OQ096423
Escherichia coli EF2 *bla*$_{TEM}$	OQ096424
Escherichia coli EF3 *bla*$_{TEM}$	OQ096425
Escherichia coli EF4 *bla*$_{TEM}$	OQ096426
Escherichia coli EM1 *bla*$_{TEM}$	OQ096419
Escherichia coli EM2 *bla*$_{TEM}$	OQ096420
Escherichia coli EM3 *bla*$_{TEM}$	OQ096421
Escherichia coli EM4 *bla*$_{TEM}$	OQ096422
Klebsiella pneumoniae KF2 *bla*$_{TEM}$	OQ096431
Klebsiella pneumoniae KF3 *bla*$_{TEM}$	OQ096432
Klebsiella pneumoniae KF4 *bla*$_{TEM}$	OQ096433
Klebsiella pneumoniae KM1 *bla*$_{TEM}$	OQ096427
Klebsiella pneumoniae KM2 *bla*$_{TEM}$	OQ096428
Klebsiella pneumoniae KM3 *bla*$_{TEM}$	OQ096429
Klebsiella pneumoniae KM4 *bla*$_{TEM}$	OQ096430
Pseudomonas aeruginosa PF3 *bla*$_{TEM}$	OQ096436
Pseudomonas aeruginosa PM3 *bla*$_{TEM}$	OQ096434
Pseudomonas aeruginosa PM4 *bla*$_{TEM}$	OQ096435

Table S3. GenBank® Accession Numbers of the reference sequences and their corresponding phylogenetic tree construction parameters. The chosen reference sequences were based on the initial Basic Local Alignment Search Tool (BLAST) analysis of the study sequences. Tree construction parameters were based on the best nucleotide substitution model identified using Akaike Information Criterion (AIC) analysis in the jModelTest version 20160303. The genes presented include 16S rRNA– 16S ribosomal ribonucleic acid, CTX–M– Cefotaximase–Munich, TEM– Temoneira β–lactamase, SHV– Sulfhydryl reagent variable β–lactamase, NDM– New Delhi metallo–β–lactamase, IMP– Imipenemase, DHA– Dharhan β–lactamase, OXA– Oxacillinase. The chosen models include GTR+G– General Time Reversible model with gamma shape, TrN– Tamura–Nei model, TrN+I– Tamura–Nei with proportion of invariant sites, HKY+G– Hasegawa–Kishino–Yano model with gamma shape, TPM1uf– Three–parameter model 1 with unequal frequencies, TPM2uf– Three–parameter model 2 with unequal frequencies, TPM2uf+I– Three–parameter model 2 with unequal frequencies and proportion of invariant sites, K80– Kimura model.

Target Gene	Reference Sequences (GenBank Accession Numbers)	Tree Construction Parameters
16S rRNA	*Klebsiella pneumoniae* (LC557135)	Model = GTR+G
	Klebsiella aerogenes (NR_102493)	Substitution rates:
	Salmonella typhimurium (DQ153191)	[AC] = 0.7468
	Citrobacter freundii (MF953251)	[AG] = 2.4750
	Escherichia coli (OP986844)	[AT] = 1.8033
	Escherichia coli (NR_024570)	[CG] = 0.8992
	Yersinia pestis (NR_025160)	[CT] = 4.5721
	Serratia liquefaciens (NR_122057)	[GT] = 1.0000
	Proteus vulgaris (NR_115878)	gamma shape = 0.3290
	Pseudomonas aeruginosa (NR_026078)	
	Pseudomonas denitrificans (MK085084)	
	Pseudomonas fluorescens (ON908465)	
	Pseudomonas syringae (NR_043716)	
	Acinetobacter johnsonii (NR_164627)	
	Acinetobacter calcoaceticus (NR_042387)	
	Acinetobacter baumannii (NR_117931)	
	Acinetobacter baumannii (NR_026206)	
$bla_{CTX–M}$	$bla_{CTX–M–1}$ (DQ915955)	Model = TrN
	$bla_{CTX–M–10}$ (AF255298)	Substitution Rates:
	$bla_{CTX–M–15}$ (JN788266)	[AC] = 1.0000
	$bla_{CTX–M–42}$ (DQ061159)	[AG] = 1.4479
	$bla_{CTX–M–64}$ (GQ300937)	[AT] = 1.0000
	$bla_{CTX–M–117}$ (JN227085)	[CG] = 1.0000
	$bla_{CTX–M–123}$ (JN790864)	[CT] = 2.6278
	$bla_{CTX–M–167}$ (KR537428)	[GT] = 1.0000
	$bla_{CTX–M–179}$ (KU705393)	
bla_{DHA}	$bla_{DHA–1}$ (JN638038)	Model = TrN+I
	$bla_{DHA–2}$ (AF259520)	Substitution Rates
	$bla_{DHA–12}$ (HG798963)	[AC] = 1.0000
	$bla_{DHA–14}$ (DQ478687)	[AG] = 3.3526
	$bla_{DHA–20}$ (KM087848)	[AT] = 1.0000
	$bla_{DHA–22}$ (KM08756)	[CG] = 1.0000
	$bla_{DHA–23}$ (KX068223)	[CT] = 7.8918
		[GT] = 1.0000

Target Gene	Reference Sequences (GenBank Accession Numbers)	Tree Construction Parameters
		proportion of invariant sites = 0.7340
bla$_{IMP}$	*bla*$_{IMP-1}$ (EF027105)<br>*bla*$_{IMP-2}$ (AJ243491)<br>*bla*$_{IMP-4}$ (AF244145)<br>*bla*$_{IMP-5}$ (AF290912)<br>*bla*$_{IMP-7}$ (AF318077)<br>*bla*$_{IMP-9}$ (FJ655384)<br>*bla*$_{IMP-11}$ (AB074436)<br>*bla*$_{IMP-12}$ (AJ420864)<br>*bla*$_{IMP-14}$ (KJ406505)<br>*bla*$_{IMP-18}$ (AY780674)	Model = HKY+I<br>kappa = 4.3430<br>transition/ transversion= 2.0136<br>proportion of invariant sites = 0.4180
bla$_{NDM}$	*bla*$_{NDM-1}$ (KJ018857)<br>*bla*$_{NDM-7}$ (KP826705)<br>*bla*$_{NDM-29}$ (MN624980)	Model = TPM1uf<br>Substitution Rates:<br>[AC] = 1.0000<br>[AG] = 1.0110<br>[AT] = 0.0100<br>[CG] = 0.0100<br>[CT] = 1.0110<br>[GT] = 1.0000
bla$_{OXA-1}$	*bla*$_{OXA-1}$ (HQ170510)<br>*bla*$_{OXA-47}$ (AY237830)<br>*bla*$_{OXA-675}$ (MH780096)	Model = HKY<br>kappa = 11.7342<br>transition/ transversion = 5.0996
bla$_{OXA-24-like}$	*bla*$_{OXA-24}$ (AY082394)<br>*bla*$_{OXA-25}$ (AF201826)<br>*bla*$_{OXA-72}$ (AY739646)<br>*bla*$_{OXA-160}$ (GU199038)	Model = TPM1uf<br>Substitution Rates:<br>[AC] = 1.0000<br>[AG] = 3.0912<br>[AT] = 2.2553<br>[CG] = 2.2553<br>[CT] = 3.0912<br>[GT] = 1.0000
bla$_{OXA-51-like}$	*bla*$_{OXA-51}$ (DQ385606)<br>*bla*$_{OXA-66}$ (AY750909)<br>*bla*$_{OXA-68}$ (AY750910)<br>*bla*$_{OXA-69}$ (AY859527)<br>*bla*$_{OXA-70}$ (AY750912)<br>*bla*$_{OXA-71}$ (AY859528)<br>*bla*$_{OXA-90}$ (EU547443)	Model = HKY+G<br>kappa = 8.1963<br>transition/ transversion = 3.9285<br>gamma shape = 0.0740
bla$_{SHV}$	*bla*$_{SHV-1}$ (AY787643)<br>*bla*$_{SHV-5}$ (EF653399)<br>*bla*$_{SHV-27}$ (AF293345)<br>*bla*$_{SHV-28}$ (AF299299)	Model = TPM2uf+I<br>Substitution Rates:<br>[AC] = 0.0100<br>[AG] = 6.7465<br>[AT] = 0.0100<br>[CG] = 1.0000<br>[CT] = 6.7465<br>[GT] = 1.0000<br>proportion of invariant sites = 0.9700
bla$_{TEM}$	*bla*$_{TEM-1}$ (AY263331)<br>*bla*$_{TEM-3}$ (FKZZ01000044)<br>*bla*$_{TEM-5}$ (NG_068215)<br>*bla*$_{TEM-20}$ (EU527189)	Model = K80<br>kappa = 2.2351<br>transition/ transversion = 1.1175

Table S4. Antibiogram profiles of the study isolates showed category–specific resistance to different antibiotics. The profiles were generated from Vitek® 2 Compact (bioMérieux, France). Resistance Category 1 (RC1; strains F1, M1) refers to the isolates non–susceptible to third–generation cephalosporins, RC2 (strains F2, M2) refers to those non–susceptible to third–generation cephalosporins and penicillin–β–lactamase inhibitors, RC3 to those resistant to carbapenems and other β–lactams (strains F3, M3), and RC4 to those with clinically unusual resistance profiles (levofloxacin–resistant but ertapenem–susceptible *Enterobacteriaceae*; carbapenem–resistant but cephalosporin–susceptible *P. aeruginosa* and *A. baumannii*, strains F4, M4). The antibiotics presented include AMC– amoxicillin–clavulanic acid, AMP– ampicillin, AMK– amikacin, CAZ– ceftazidime, CIP– ciprofloxacin, CTR– ceftriaxone, CFX– cefuroxime, ERT– ertapenem, CEF– cefepime, NIT– nitrofurantoin, GEN– gentamicin, IMI– imipenem, LEV– levofloxacin, MEM– meropenem, SX – trimethoprim–sulfamethoxazole, TZP– piperacillin–tazobactam. The profiles presented are based on the latest Clinical and Laboratory Standards Institute breakpoints and recommendations: S– susceptible, I– intermediate, R– resistant, NT– not tested.

	AMC	AMP	AMK	CAZ	CIP	CTR	CFX	ERT	CEF	NIT	GEN	IMI	LEV	MEM	SXT	TZP
Klebsiella pneumoniae																
KF1	S	R	S	R	R	R	R	S	R	NT	S	S	S	S	S	S
KF2	R	R	S	R	R	R	R	S	R	NT	S	S	S	S	R	I
KF3	R	R	S	R	R	R	R	R	R	NT	R	R	R	R	R	R
KF4	S	R	S	R	R	R	R	S	R	NT	S	S	R	S	S	S
KM1	S	R	S	R	I	R	R	S	R	NT	S	S	S	S	S	S
KM2	S	R	S	R	S	R	R	S	R	NT	S	S	I	S	S	I
KM3	R	R	R	R	R	R	R	R	R	NT	R	R	R	R	R	R
KM4	S	R	S	R	R	R	R	S	R	NT	S	S	R	S	S	S
Escherichia coli																
EF1	S	R	S	R	S	R	R	S	R	NT	S	S	S	S	S	S
EF2	R	R	NT	R	S	R	R	S	R	NT	S	S	S	S	S	S
EF3	R	R	S	R	R	R	R	R	R	S	S	R	R	R	R	R
EF4	S	R	S	R	R	R	R	S	R	S	S	S	R	S	S	S
EM1	S	R	S	R	I	R	R	S	R	NT	S	S	NT	S	S	S
EM2	R	R	S	R	R	R	R	S	R	S	R	S	R	S	S	I
EM3	R	R	S	R	R	R	R	S	R	S	R	R	R	R	R	R
EM4	S	R	S	R	R	R	R	S	R	S	S	S	R	S	R	S
Acinetobacter baumannii																
AF1	NT	NT	NT	S	R	I	NT	NT	I	NT	S	S	NT	S	S	S
AF2	I	NT	S	I	S	I	NT	NT	I	NT	S	S	S	S	S	S
AF3	R	NT	NT	R	R	R	NT	NT	R	NT	R	R	NT	R	R	R
AF4	NT	NT	S	S	S	I	NT	NT	S	NT	S	R	S	R	S	S
AM1	NT	NT	NT	R	R	R	NT	NT	R	NT	R	S	NT	S	R	S
AM2	R	NT	S	S	S	I	NT	NT	S	NT	S	S	S	S	R	S
AM3	R	NT	S	R	R	R	NT	NT	R	NT	R	R	NT	R	R	R
Pseudomonas aeruginosa																
PF2	NT	NT	S	R	S	NT	NT	NT	I	NT	S	S	S	S	NT	R
PF3	NT	NT	S	R	R	NT	NT	NT	R	NT	R	R	R	R	NT	R
PF4	NT	NT	S	S	S	NT	NT	NT	S	NT	I	R	S	R	NT	S
PM2	NT	NT	S	R	S	NT	NT	NT	R	NT	S	S	S	S	NT	R
PM3	NT	NT	R	R	R	NT	NT	NT	R	NT	R	R	R	R	NT	R
PM4	NT	NT	S	S	S	NT	NT	NT	S	NT	S	R	S	R	NT	I

Table S5. Results of the disk diffusion–based phenotypic assays for detecting extended–spectrum β–lactamase (ESBL) and carbapenemase (CP) among the study isolates showed the production of ESBLs in the majority of the isolates, and CPs in most RC3 isolates (KF3, KM3, EF3, EM3, AF3, AM3, PF3, PM3). All tests are based on the latest Clinical and Laboratory Standards Institute protocols. For ESBL testing, the antibiotic disks used include CAC– ceftazidime–clavulanic acid (30 µg/10 µg), CAZ– ceftazidime (30 µg), CEC– cefotaxime–clavulanic acid (30 µg/10 µg), CTX– cefotaxime (30 µg). The values presented under this test are the average differences between the zones of inhibitions (ZOI in millimeters mm) of the combination disks and the standalone cephalosporin disks in triplicate setups. A difference of at least 5mm in ZOI in either CAC–CAZ or CEC–CTX indicated ESBL production (+), otherwise, the isolate was reported to be a non–ESBL producer (–). CP production was detected using modified carbapanem inactivation (mCIM) and ethylenediamene tetraacetic acid (EDTA)–modified carbapenem inactivation (eCIM) methods. The antibiotic disk used is 10µg meropenem. The values presented are the average ZOI of the disk (in mm) in triplicate setups. A 6–15 mm ZOI (or 16–18 mm ZOI with pinpoint colonies within the zones) in the mCIM setups is indicated putative serine CP production (+). For all mCIM positive isolates, at least 5 mm increase in the ZOI from the mCIM to the eCIM setups indicated that the CP produced is a metallo–β–lactamase (++). Isolates not confirming with mCIM criteria (regardless of eCIM results) are reported as non–CP producers (–).

Organism	ESBL Testing			mCIM and eCIM Testing		
	CAC–CAZ (mm)	CEC–CTX (mm)	ESBL	mCIM (mm)	eCIM (mm)	CP
K. pneumoniae KF1	9.3	16.33	+	0	0	–
K. pneumoniae KF2	2.2	24.67	+	21.67	24.67	–
K. pneumoniae KF3	0	8.5	+	6	23.83	++
K. pneumoniae KF4	21.33	14.17	+	22.67	23.67	–
K. pneumoniae KM1	10.67	9.67	+	22	22.33	–
K. pneumoniae KM2	26.17	16.33	+	21.67	21	–
K. pneumoniae KM3	0	9.67	+	6	20.67	++
K. pneumoniae KM4	12.5	16.33	+	24.33	21.67	–
E. coli EF1	6.33	15.61	+	20.67	20	–
E. coli EF2	–0.75	–0.5	–	18.67	21.33	–
E. coli EF3	0	10	+	10.67	20.33	++
E. coli EF4	4.66	13.83	+	20.67	20.67	–
E. coli EM1	8.5	16.17	+	21.67	23	–
E. coli EM2	28.67	32.17	+	20.67	25.33	–
E. coli EM3	0	18.33	+	11.33	17.33	++
E. coli EM4	6	–6.33	+	19.67	20	–
A. baumannii AF1	–1.5	3.33	–	22.33	23	–
A. baumannii AF2	0	4	–	23.33	23.67	–
A. baumannii AF3	0	4	–	20	20	–
A. baumannii AF4	–0.33	1.17	–	25	24	–
A. baumannii AM1	4.83	22.17	+	20.33	20.67	–
A. baumannii AM2	–1	2.67	–	19.33	20	–
A. baumannii AM3	–1.33	2.33	–	16.33	18	+
P. aeruginosa PF2	1	9	+	22.67	22	–
P. aeruginosa PF3	0	13.67	+	22	0	+
P. aeruginosa PF4	0.5	6.17	+	25	24.5	–
P. aeruginosa PM2	0.5	10.33	+	21.33	21.67	–
P. aeruginosa PM3	2.67	14.83	+	23	23	–
P. aeruginosa PM4	1.33	9.5	+	22.87	23	–

Table S6. Results of the Xpert® Carba–R PCR (Cepheid, California, USA) revealed the production of bla_{NDM} in some test isolates. The genes tested using the kit include IMP– imipenemase, VIM– Verona integron–encoded metallo–β–lactamase, NDM– New Delhi metallo–β–lactamase, KPC– *K. pneumoniae* carbapenemase, and OXA–48– oxacillinase–48. The presence (+) or absence (–) of the genes was reported qualitatively by GeneXpert® IV System (Cepheid, California, USA). *K. pneumoniae* F1 was excluded due to invalid results.

Organism	Target Genes				
	bla_{IMP}	bla_{VIM}	bla_{NDM}	bla_{KPC}	$bla_{OXA–48}$
K. pneumoniae KF2	–	–	–	–	–
K. pneumoniae KF3	–	–	+	–	–
K. pneumoniae KF4	–	–	–	–	–
K. pneumoniae KM1	–	–	–	–	–
K. pneumoniae KM2	–	–	–	–	–
K. pneumoniae KM3	–	–	+	–	–
K. pneumoniae KM4	–	–	–	–	–
E. coli EF1	–	–	–	–	–
E. coli EF2	–	–	–	–	–
E. coli EF3	–	–	+	–	–
E. coli EF4	–	–	–	–	–
E. coli EM1	–	–	–	–	–
E. coli EM2	–	–	–	–	–
E. coli EM3	–	–	–	–	–
E. coli EM4	–	–	–	–	–
A. baumannii AF1	–	–	–	–	–
A. baumannii AF2	–	–	–	–	–
A. baumannii AF3	–	–	–	–	–
A. baumannii AF4	–	–	–	–	–
A. baumannii AM1	–	–	–	–	–
A. baumannii AM2	–	–	–	–	–
A. baumannii AM3	–	–	–	–	–
P. aeruginosa PF2	–	–	–	–	–
P. aeruginosa PF3	–	–	–	–	–
P. aeruginosa PF4	–	–	–	–	–
P. aeruginosa PM2	–	–	–	–	–
P. aeruginosa PM3	–	–	–	–	–
P. aeruginosa PM4	–	–	–	–	–

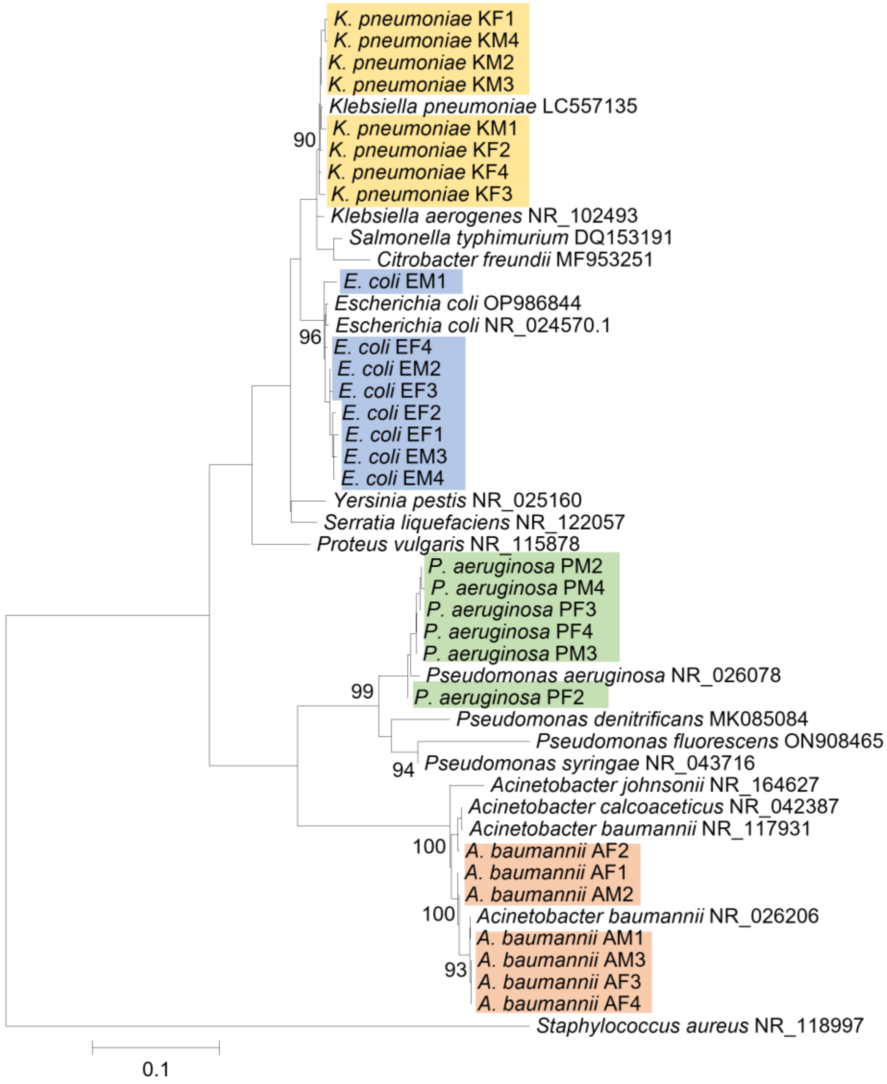

Figure S1. Neighbor–joining tree of the study isolates (colored entries) and reference species based on 694 nucleotides of 16S rRNA gene using the General Time Reversible with gamma distribution (GTR+G) model of DNA substitution. The tree is rooted in *Staphylococcus aureus*. Numbers on the nodes represent bootstrap values out of 1000 replicates. Values less than 90% are not shown. The scale bar represents 10 substitutions for every 100 nucleotides. General tree topology and bootstrap probability values confirm the identities of the study isolates as *Klebsiella pneumoniae* (90% bootstrap value in *K. pneumoniae* reference clade), *Escherichia coli* (96% bootstrap value in *E. coli* reference clade), *Pseudomonas aeruginosa* (99% bootstrap value in *P. aeruginosa* reference clade), and *Acinetobacter baumannii* (100% bootstrap value in *A. baumannii* reference clade).

A.

B.

C.

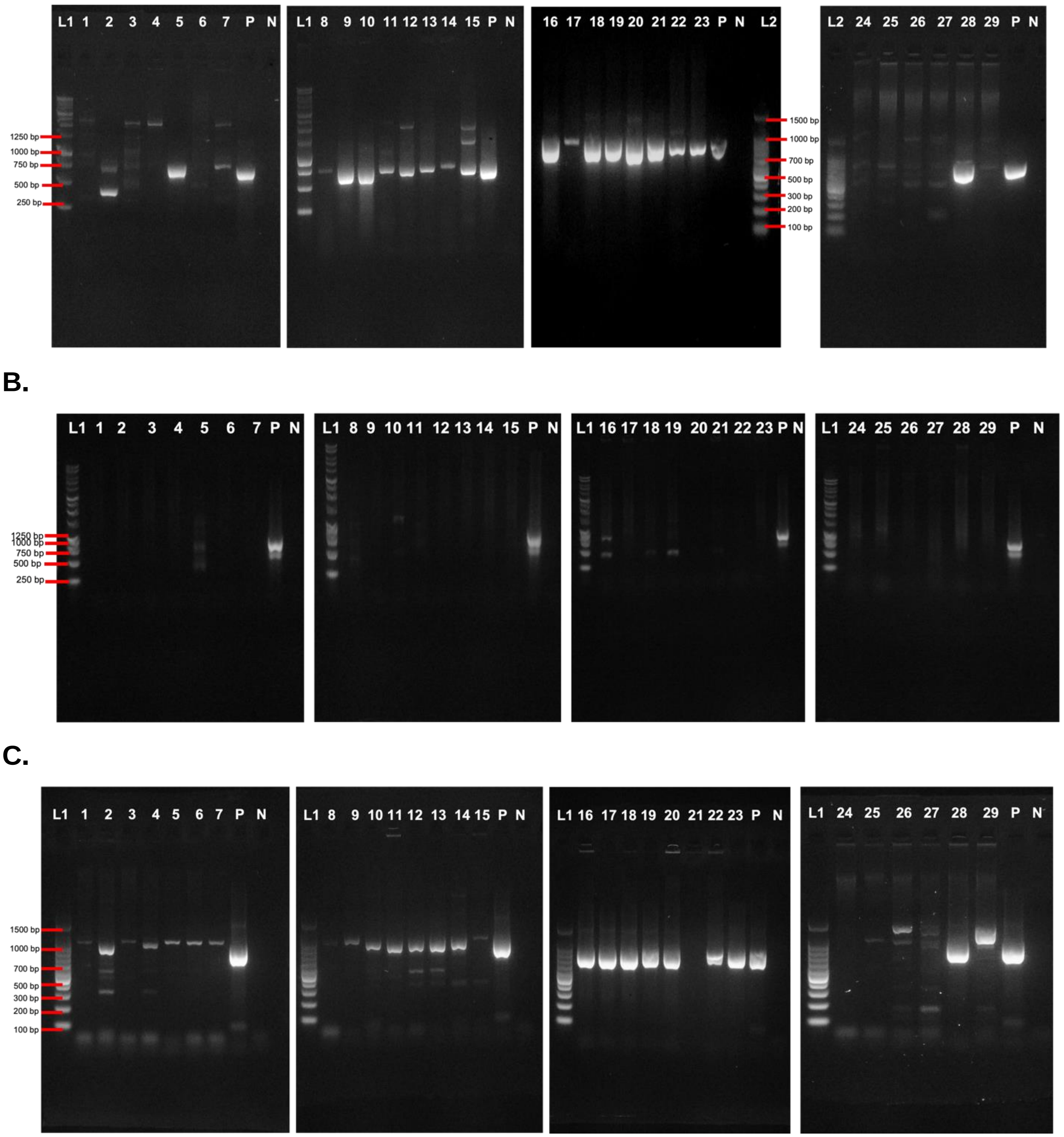
L1 1 2 3 4 5 6 7 P N
1250 bp
1000 bp
750 bp
500 bp
250 bp
L1 8 9 10 11 12 13 14 15 P N
16 17 18 19 20 21 22 23 P N L2
1500 bp
1000 bp
700 bp
500 bp
300 bp
200 bp
100 bp
L2 24 25 26 27 28 29 P N
L1 16 17 18 19 20 21 22 23 P N
L1 24 25 26 27 28 29 P N
1500 bp
1000 bp
700 bp
500 bp
300 bp
200 bp
100 bp

D.

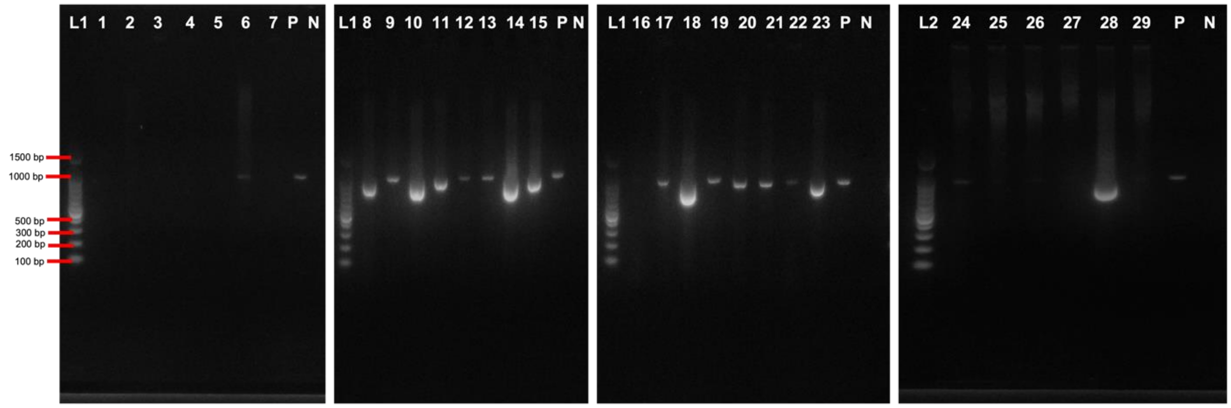

Figure S2. Agarose gel electrophoresis profiles of the PCR amplicons for the tested Ambler Class A β–lactamases**.** For convenience, the images of these four gels were assembled using Microsoft PowerPoint and the numbering of the lanes of the four gels was continuous (1 through 29). Gel exposure was optimized in E–Gel™ Power Snap imaging system (Thermo Fisher Scientific, USA). Gel contrast and brightness were adjusted using Microsoft Powerpoint to improve band clarity. **A.** Agarose gel electrophoresis profiles of the *bla*$_{CTX–M}$ amplicons. The expected amplicon band size is 688 bp. Lanes 1–7 (Lanes 1–4 *A. baumannii* AF1–AF4, Lanes 5–7 – *A. baumannii* AM1–AM3), Lanes 8–15 (Lanes 8–11, *E. coli* EF1–EF4, Lanes 12–15 – *E. coli* EM1–EM4), Lanes 16–23 (Lanes 16–19 *K. pneumoniae* KF1–KF4, Lanes 20–23 *K. pneumoniae* KM1–KM4). Lanes 24–29 (Lanes 24–26 *P. aeruginosa* PF2–PF4, Lanes 27–29 *P. aeruginosa* PM2–PM4). L1 – 1 Kbp DNA Ladder. L2 – 100 bp DNA Ladder. P – Positive Control (*E. coli* NCTC 13353). N – Negative Control (*S. aureus* ATCC 29213). **B.** Agarose gel electrophoresis profiles of the *bla*$_{KPC}$ amplicons. The expected amplicon band size is 882 bp. Lanes 1–7 (Lanes 1–3 *A. baumannii* AM1–AM3, Lanes 4–7 *A. baumannii* AF1–AF4). Lanes 8–15 (Lanes 8–11 *E. coli* EM1–EM4, Lanes 12–15 *E. coli* EF1–EF4). Lanes 16–23 (Lanes 16–19 *K. pneumoniae* KM1–KM4, Lanes 20–23 *K. pneumoniae* KF1–KF4). Lanes 24–29 (Lanes 24–26 *P. aeruginosa* PM2–PM4, Lanes 27–29 *P. aeruginosa* PF2–PF4). L1 – 1 Kbp DNA Ladder. P – Positive Control (*K. pneumoniae* ATCC BAA 1705). N – Negative Control (*S. aureus* ATCC 29213). **C.** Agarose gel electrophoresis profiles of the *bla*$_{SHV}$ amplicons. The expected amplicon band size is 797 bp. Lanes 1–7 (Lanes 1–3 *A. baumannii* AM1–AM3, Lanes 4–7 *A. baumannii* AF1–AF4). Lanes 8–15 (Lanes 8–11 *E. coli* EM1–EM4, Lanes 12–15 *E. coli* EF1–EF4). Lanes 16–23 (Lanes 16–19 *K. pneumoniae* KM1–KM4, Lanes 20–23 *K. pneumoniae* KF1–KF4). Lanes 24–29 (Lanes 24–26 *P. aeruginosa* PM2–PM4, Lanes 27–29 *P. aeruginosa* PF2–PF4). L1 – 100 bp DNA Ladder. P – Positive Control (*K. pneumoniae* ATCC 700603). N – Negative Control (*S. aureus* ATCC 29213). Lane numbers 1 and 6 showed presence of putative target amplicons, but sequencing revealed mixed or not high–quality chromatogram signals. These sequences were not used in the analyses. Lane numbers 8 to 15 had low quality sequences with matching 24 to 30 bp only in references. **D.** Agarose gel electrophoresis profiles of the *bla*$_{TEM}$ amplicons. The expected amplicon band size is 800 bp. Lanes 1–7 (Lanes 1–4 *A. baumannii* AF1–AF4, Lane 5–7 – *A. baumannii* AM1–AM3), Lanes 8–15 (Lanes 8–11, *E. coli* EF1–EF4, Lanes 12–15 – *E. coli* EM1–EM4), Lanes 16–23 (Lanes 16–19 *K. pneumoniae* KF1–KF4, Lanes 20–23 *K. pneumoniae* KM1–KM4). Lanes 24–29 (Lanes 24–26 *P. aeruginosa* PF2–PF4, Lanes 27–29 *P. aeruginosa* PM2–PM4). L1 – 100 bp DNA Ladder. P – Positive Control (*E. coli* NCTC 13353). N – Negative Control (*S. aureus* ATCC 29213). All putative amplicons of the gene targets were sequenced to confirm gene identities. Only the sequence–confirmed PCR amplicons were used in the analysis. The genes presented include CTX–M– Cefotaximase–Munich, KPC– *K. pneumoniae* carbapenemase, SHV– Sulfhydryl reagent variable β–lactamase, and TEM– Temoneira β–lactamase.

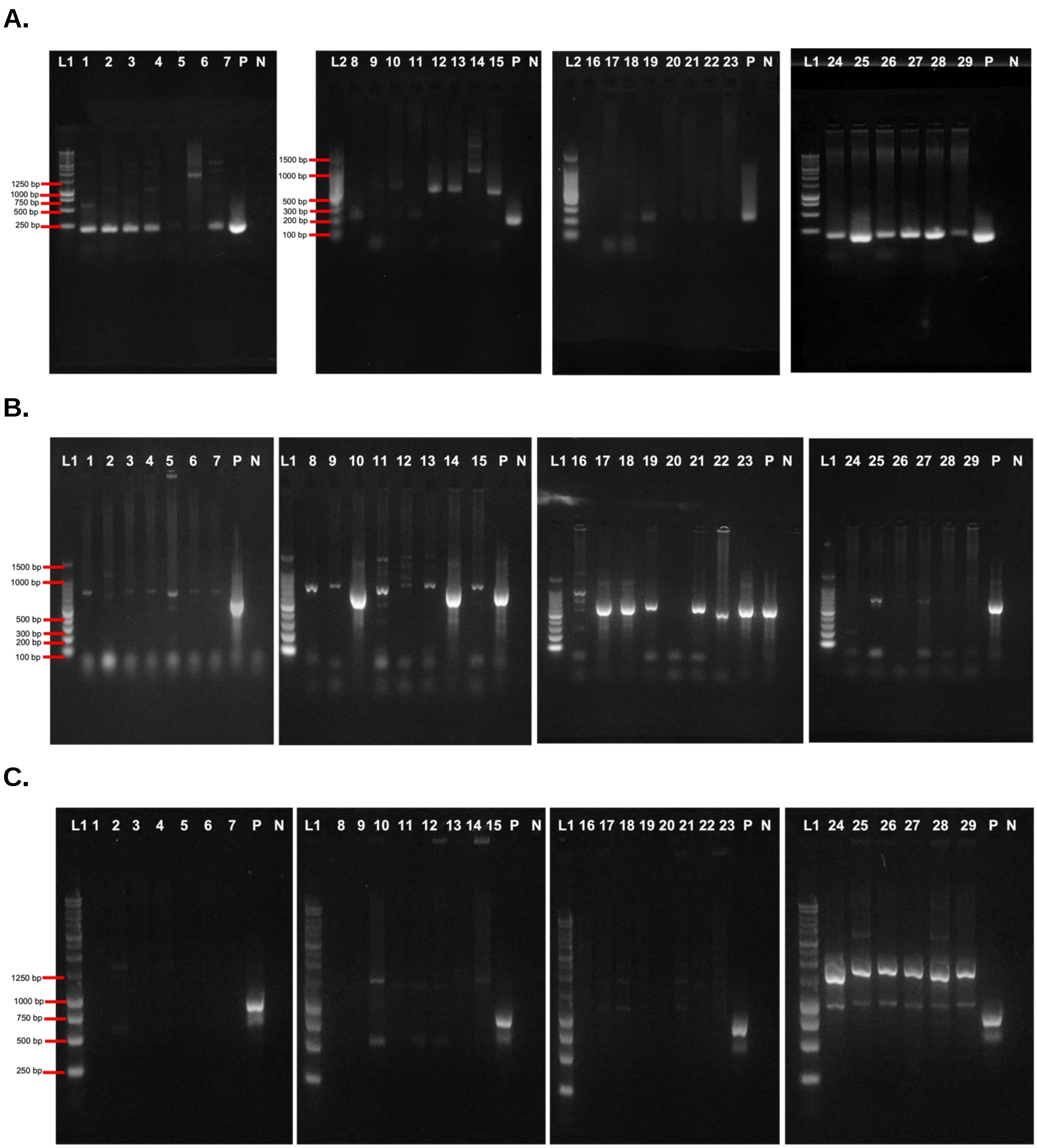

Figure S3. Agarose gel electrophoresis profiles of the PCR amplicons for the tested Ambler Class B β–lactamases. For convenience, the images of these four gels were assembled using Microsoft PowerPoint and the numbering of the lanes of the four gels was continuous (1 through 29). Gel exposure was optimized in E–Gel™ Power Snap imaging system (Thermo Fisher Scientific, USA). Gel contrast and brightness were adjusted using Microsoft PowerPoint to improve band clarity. **A**. Agarose gel electrophoresis profiles of the bla_{IMP} amplicons. The expected amplicon

band size is 233 bp. Lanes 1–7 (Lanes 1–4 *A. baumannii* AF1–AF4, Lanes 5–7 – *A. baumannii* AM1–AM3), Lanes 8–15 (Lanes 8–11, *E. coli* EF1–EF4, Lanes 12–15 – *E. coli* EM1–EM4), Lanes 16–23 (Lanes 16–19 *K. pneumoniae* KF1–KF4, Lanes 20–23 *K. pneumoniae* KM1–KM4). Lanes 24–29 (Lanes 24–26 *P. aeruginosa* PF2–PF4, Lanes 27–29 *P. aeruginosa* PM2–PM4). L1 – 1 Kbp DNA Ladder. L2 – 100 bp DNA Ladder. P – Positive Control (*E. coli* NCTC 13476). N – Negative Control (*S. aureus* ATCC 29213). Lane number 6 had low quality sequence with matching 20 bp only in reference. **B.** Agarose gel electrophoresis profiles of the bla_{NDM} amplicons. The expected amplicon band size is 621 bp. Lanes 1–7 (Lanes 1–3 *A. baumannii A*M1–AM3, Lanes 4–7 *A. baumannii* AF1–AF4). Lanes 8–15 (Lanes 8–11 *E. coli* M1–M4, Lanes 12–15 *E. coli* EF1–EF4). Lanes 16–23 (Lanes 16–19 *K. pneumoniae K*M1–KM4, Lanes 20–23 *K. pneumoniae* KF1–KF4). Lanes 24–29 (Lanes 24–26 *P. aeruginosa P*M2–PM4, Lanes 27–29 *P. aeruginosa P*F2–PF4). L1 – 100 bp DNA Ladder P – Positive Control (*K. pneumoniae* ATCC BAA 2146). N – Negative Control (*S. aureus* ATCC 29213). Lane number 22 had low quality sequence with matching 20 bp only in reference. **C.** Agarose gel electrophoresis profiles of the bla_{VIM} amplicons. The expected amplicon band size is 801 bp. Lanes 1–7 (Lanes 1–3 *A. baumannii A*M1–AM3, Lanes 4–7 *A. baumannii* AF1–AF4). Lanes 8–15 (Lanes 8–11 *E. coli E*M1–EM4, Lanes 12–15 *E. coli* EF1–EF4). Lanes 16–23 (Lanes 16–19 *K. pneumoniae K*M1–KM4, Lanes 20–23 *K. pneumoniae* KF1–KF4). Lanes 24–29 (Lanes 24–26 *P. aeruginosa P*M2–PM4, Lanes 27–29 *P. aeruginosa P*F2–PF4). L1 – 1 Kbp DNA Ladder. P – Positive Control (*K. pneumoniae* NCTC BAA 13439). N – Negative Control (*S. aureus* ATCC 29213). All putative amplicons of the gene targets were sequenced to confirm gene identities. Only the sequence–confirmed PCR amplicons were used in the analysis. The genes presented include IMP– Imipenemase, NDM– New Delhi metallo–β–lactamase, and VIM– Verona integron–encoded metallo–β–lactamase.

A.

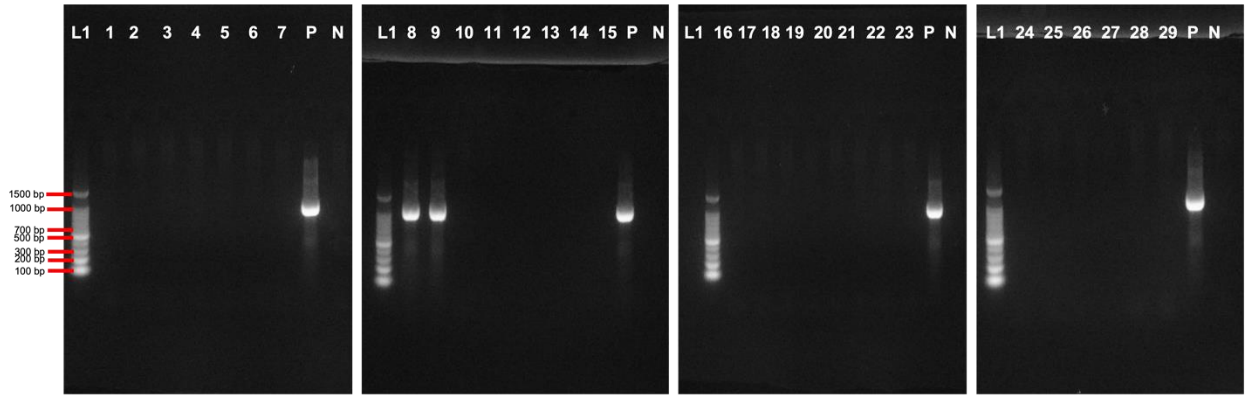

B.

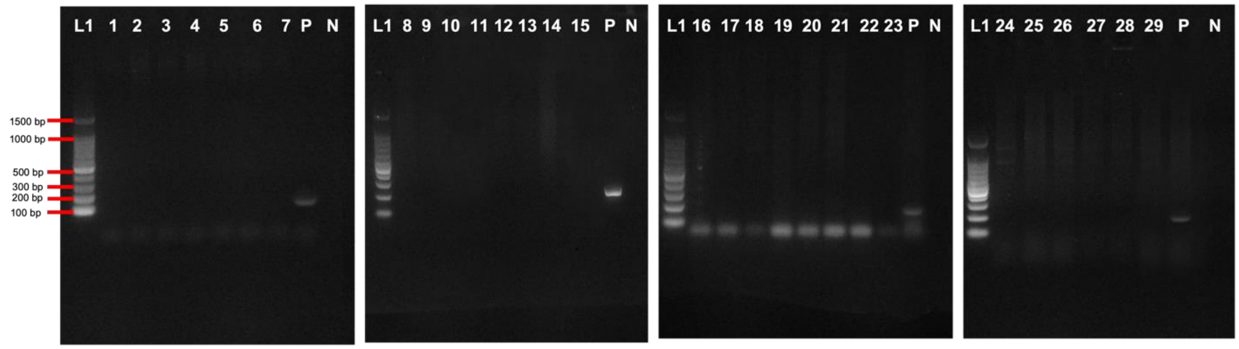

Figure S4. Agarose gel electrophoresis profiles of the PCR amplicons for the tested Ambler Class C β–lactamases. For convenience, the images of these four gels were assembled using Microsoft PowerPoint and the numbering of the lanes of the four gels was continuous (1 through 29). Gel exposure was optimized in E–Gel™ Power Snap imaging system (Thermo Fisher Scientific, USA). Gel contrast and brightness were adjusted using Microsoft PowerPoint to improve band clarity. **A**. Agarose gel electrophoresis profiles of the *bla*$_{DHA}$ amplicons. The expected amplicon band size is 997 bp. Lanes 1–7 (Lanes 1–4 *A. baumannii* AF1–AF4, Lane 5–7 – *A. baumannii* AM1–AM3), Lanes 8–15 (Lanes 8–11, *E. coli* EF1–EF4, Lanes 12–15 – *E. coli* EM1–EM4), Lanes 16–23 (Lanes 16–19 *K. pneumoniae* KF1–KF4, Lanes 20–23 *K. pneumoniae* KM1–KM4). Lanes 24–29 (Lanes 24–26 *P. aeruginosa* PF2–PF4, Lanes 27–29 *P. aeruginosa* PM2–PM4). L1 – 100 bp DNA Ladder. P – Positive Control (*K. pneumoniae* ATCC 2146). N – Negative Control (*S. aureus* ATCC 29213). **B.** Agarose gel electrophoresis profiles of the *bla*$_{FOX}$ amplicons. The expected amplicon band size is 190 bp. Lanes 1–7 (Lanes 1–4 *A. baumannii* AF1–AF4, Lane 5–7 – *A. baumannii* AM1–AM3), Lanes 8–15 (Lanes 8–11, *E. coli* EF1–EF4, Lanes 12–15 – *E. coli* EM1–EM4), Lanes 16–23 (Lanes 16–19 *K. pneumoniae* KF1–KF4, Lanes 20–23 *K. pneumoniae* KM1–KM4). Lanes 24–29 (Lanes 24–26 *P. aeruginosa* PF2–PF4, Lanes 27–29 *P. aeruginosa* PM2–PM4). L1 – 100 bp DNA Ladder. All putative amplicons of the gene targets were sequenced to confirm gene identities. Only the sequence–confirmed PCR amplicons were used in the analysis. The genes presented include DHA– Dharhan β–lactamase, and FOX– cefoxitin–active β–lactamase.

A.

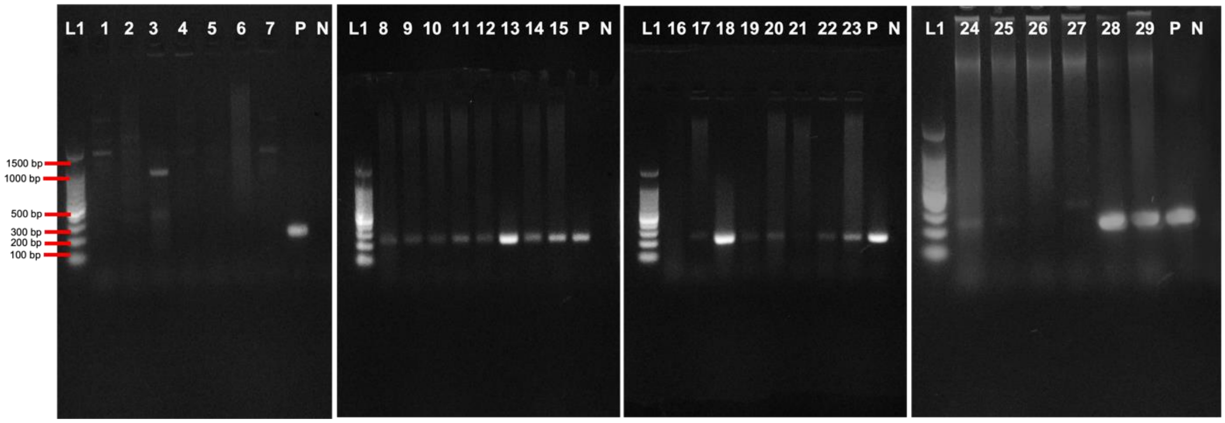
L1 1 2 3 4 5 6 7 P N
L1 8 9 10 11 12 13 14 15 P N
L1 16 17 18 19 20 21 22 23 P N
L1 24 25 26 27 28 29 P N
1500 bp
1000 bp
500 bp
300 bp
200 bp
100 bp

B.

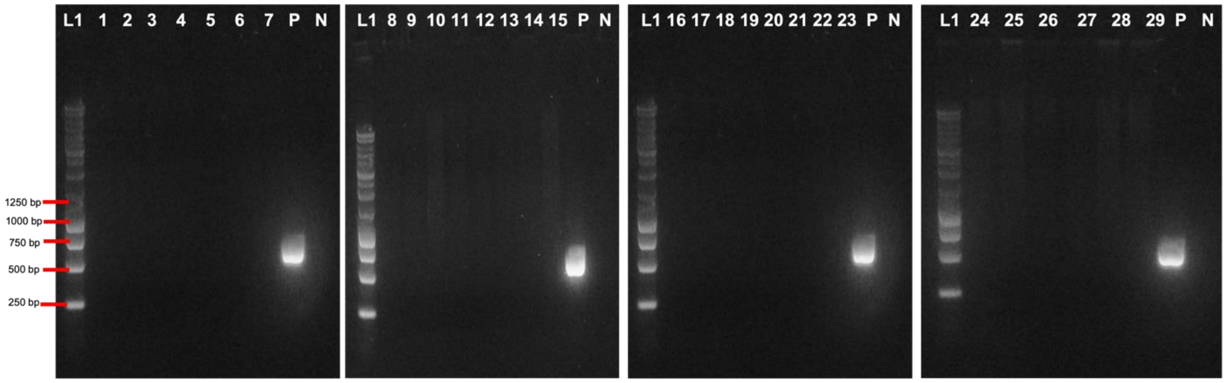
L1 1 2 3 4 5 6 7 P N
L1 8 9 10 11 12 13 14 15 P N
L1 16 17 18 19 20 21 22 23 P N
L1 24 25 26 27 28 29 P N
1250 bp
1000 bp
750 bp
500 bp
250 bp

C.

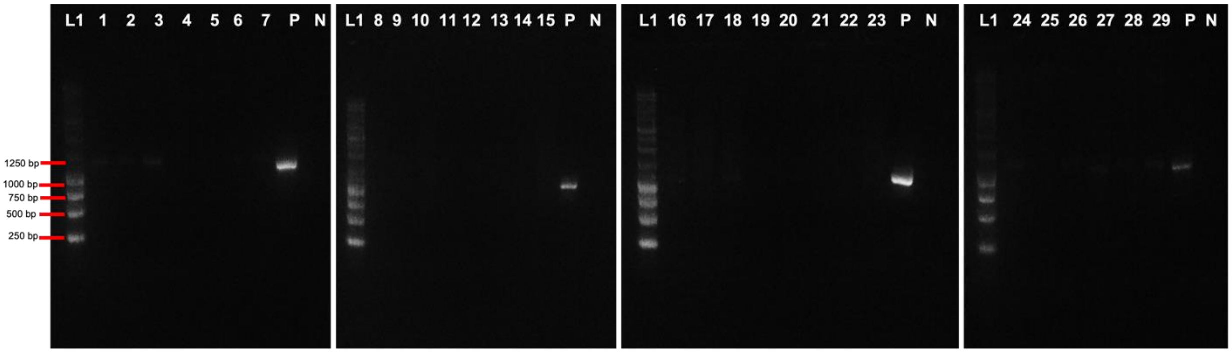
L1 1 2 3 4 5 6 7 P N
L1 8 9 10 11 12 13 14 15 P N
L1 16 17 18 19 20 21 22 23 P N
L1 24 25 26 27 28 29 P N
1250 bp
1000 bp
750 bp
500 bp
250 bp

D.

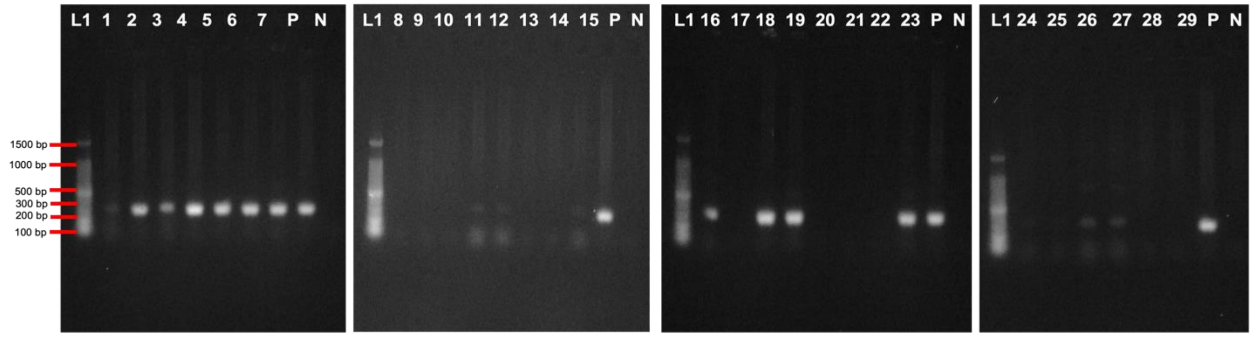
L1 1 2 3 4 5 6 7 P N
L1 8 9 10 11 12 13 14 15 P N
L1 16 17 18 19 20 21 22 23 P N
L1 24 25 26 27 28 29 P N
1500 bp
1000 bp
500 bp
300 bp
200 bp
100 bp

E.

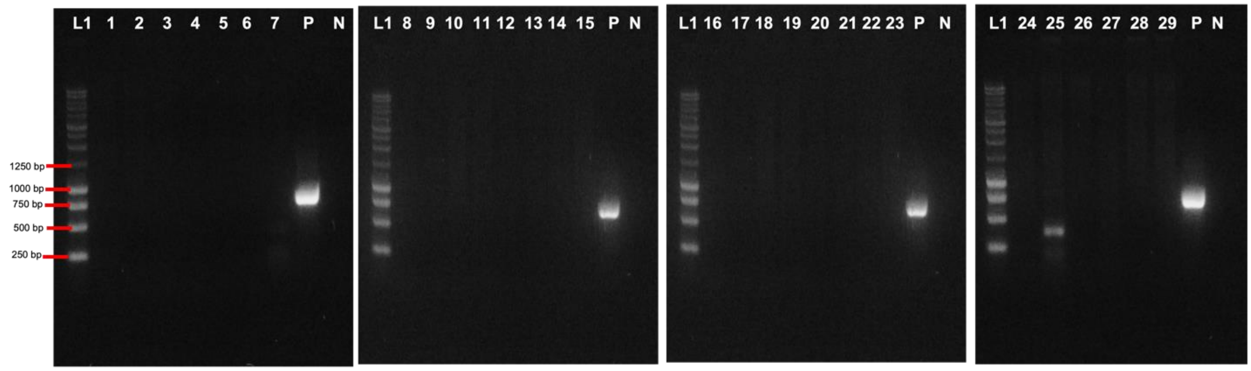
L1 1 2 3 4 5 6 7 P N
L1 8 9 10 11 12 13 14 15 P N
L1 16 17 18 19 20 21 22 23 P N
L1 24 25 26 27 28 29 P N
1250 bp
1000 bp
750 bp
500 bp
250 bp

F.

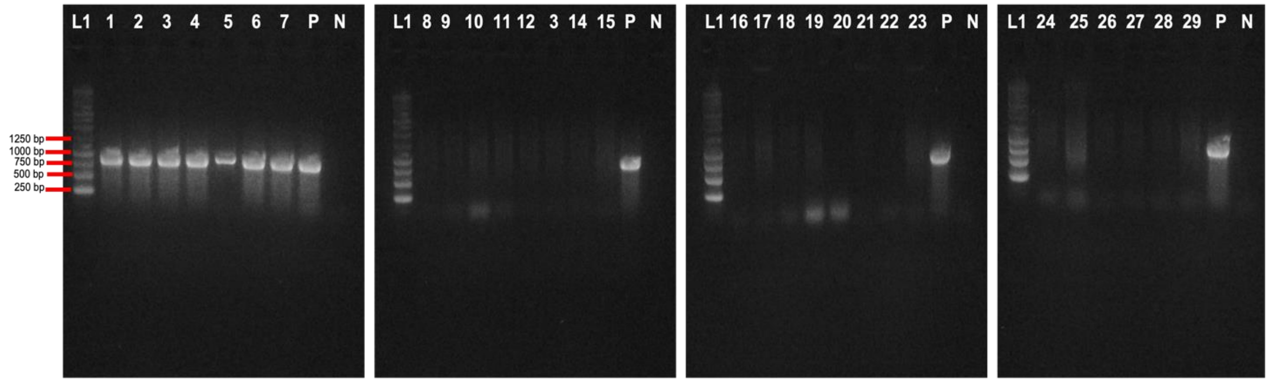
L1 1 2 3 4 5 6 7 P N
L1 8 9 10 11 12 3 14 15 P N
L1 16 17 18 19 20 21 22 23 P N
L1 24 25 26 27 28 29 P N
1250 bp
1000 bp
750 bp
500 bp
250 bp

G.

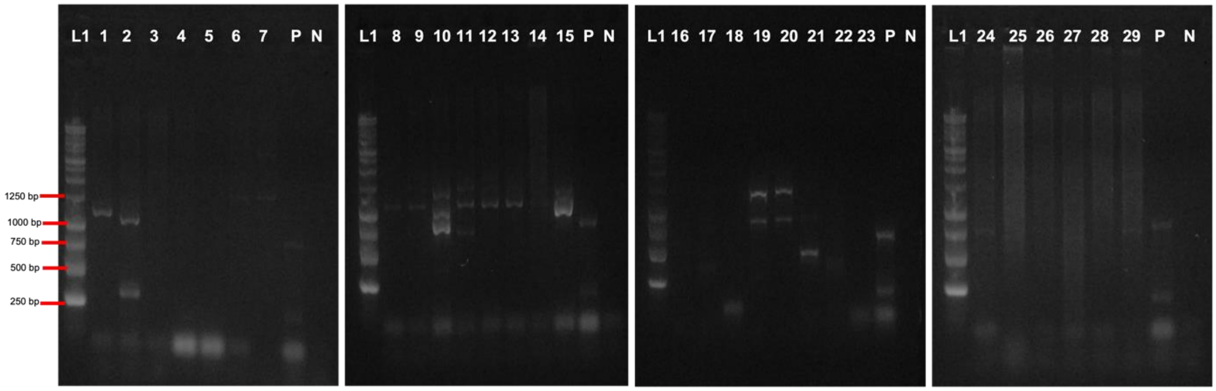

Figure S5. Agarose gel electrophoresis profiles of the PCR amplicons for the tested Ambler Class B β–lactamases. For convenience, the images of these four gels were assembled using Microsoft PowerPoint and the numbering of the lanes of the four gels was continuous (1 through 29). Gel exposure was optimized in E–Gel™ Power Snap imaging system (Thermo Fisher Scientific, USA). Gel contrast and brightness were adjusted using Microsoft PowerPoint to improve band clarity. **A**. Agarose Gel Electrophoresis of the *bla*$_{OXA-1}$ amplicons. The expected amplicon band size is 245 bp. Lanes 1–7 (Lanes 1–3 *A. baumannii A*M1–AM3, Lanes 4–7 *A. baumannii* AF1–AF4). Lanes 8–15 (Lanes 8–11 *E. coli E*M1–EM4, Lanes 12–15 *E. coli* EF1–EF4). Lanes 16–23 (Lanes 16–19 *K. pneumoniae K*M1–KM4, Lanes 20–23 *K. pneumoniae* KF1–KF4). Lanes 24–29 (Lanes 24–26 *P. aeruginosa P*M2–PM4, Lanes 27–29 *P. aeruginosa P*F2–PF4). L1 – 1 Kbp DNA Ladder. P – Positive Control (*E. coli* ATCC BAA 2469). N – Negative Control (*S. aureus* ATCC 29213). **B.** Agarose gel electrophoresis profiles of the *bla*$_{OXA-2}$ amplicons. 569 bp. Lanes 1–7 (Lanes 1–4 *A. baumannii A*F1–AF4, Lane 5–7 – *A. baumannii* AM1–AM3), Lanes 8–15 (Lanes 8–11, *E. coli E*F1–EF4, Lanes 12–15 – *E. coli* EM1–EM4), Lanes 16–23 (Lanes 16–19 *K. pneumoniae K*F1–KF4, Lanes 20–23 *K. pneumoniae* KM1–KM4). Lanes 24–29 (Lanes 24–26 *P. aeruginosa P*F2–PF4, Lanes 27–29 *P. aeruginosa P*M2–PM4). L1 – 1 Kbp DNA Ladder. P – Positive Control (*K. pneumoniae* ATCC 700603). N – Negative Control (*S. aureus* ATCC 29213). **C.** Agarose gel electrophoresis profiles of the *bla*$_{OXA-23}$ amplicons. The expected amplicon band size is 1 Kbp. Lanes 1–7 (Lanes 1–3 *A. baumannii A*M1–AM3, Lanes 4–7 *A. baumannii* AF1–AF4). Lanes 8–15 (Lanes 8–11 *E. coli* AM1–AM4, Lanes 12–15 *E. coli* AF1–AF4). Lanes 16–23 (Lanes 16–19 *K. pneumoniae* KM1–KM4, Lanes 20–23 *K. pneumoniae* KF1–KF4). Lanes 24–29 (Lanes 24–26 *P. aeruginosa* PM2–PM4, Lanes 27–29 *P. aeruginosa* PF2–PF4). L1 – 100 bp DNA Ladder. P – Positive Control (*A. baumannii* ATCC BAA–1605). N – Negative Control (*S. aureus* ATCC 29213). **D.** Agarose gel electrophoresis profiles of the *bla*$_{OXA-24-like}$ amplicons. The expected amplicon band size is 250 bp. Lanes 1–7 (Lanes 1–3 *A. baumannii* AM1–AM3, Lanes 4–7 *A. baumannii* AF1–AF4). Lanes 8–15 (Lanes 8–11 *E. coli* EM1–EM4, Lanes 12–15 *E. coli* EF1–EF4). Lanes 16–23 (Lanes 16–19 *K. pneumoniae* KM1–KM4, Lanes 20–23 *K. pneumoniae* KF1–KF4). Lanes 24–29 (Lanes 24–26 *P. aeruginosa* PM2–PM4, Lanes 27–29 *P. aeruginosa* PF2–PF4). L1 – 100 bp DNA Ladder. P – Positive Control (*A. baumannii* ATCC BAA–1605). N – Negative Control (*S. aureus* ATCC 29213). **E.** Agarose gel electrophoresis profiles of the *bla*$_{OXA-48}$ amplicons. The expected amplicon band size is 744 bp. Lanes 1–7 (Lanes 1–3 *A. baumannii* AM1–AM3, Lanes 4–7 *A. baumannii* AF1–AF4). Lanes 8–15 (Lanes 8–11 *E. coli* EM1–EM4,

Lanes 12–15 *E. coli* EF1–EF4). Lanes 16–23 (Lanes 16–19 *K. pneumoniae* KM1–KM4, Lanes 20–23 *K. pneumoniae* KF1–KF4). Lanes 24–29 (Lanes 24–26 *P. aeruginosa* PM2–PM4, Lanes 27–29 *P. aeruginosa* PF2–PF4). L1 – 1 Kbp DNA Ladder. P – Positive Control (*K. pneumoniae* NCTC 13442). N – Negative Control (*S. aureus* ATCC 29213). **F.** Agarose gel electrophoresis profiles of the $bla_{OXA-51-like}$ amplicons. The expected amplicon band size is 825 bp. Lanes 1–7 (Lanes 1–3 *A. baumannii A*M1–AM3, Lanes 4–7 *A. baumannii* AF1–AF4). Lanes 8–15 (Lanes 8–11 *E. coli E*M1–EM4, Lanes 12–15 *E. coli* EF1–EF4). Lanes 16–23 (Lanes 16–19 *K. pneumoniae K*M1–KM4, Lanes 20–23 *K. pneumoniae* KF1–KF4). Lanes 24–29 (Lanes 24–26 *P. aeruginosa P*M2–PM4, Lanes 27–29 *P. aeruginosa P*F2–PF4). L1 – 1 Kbp DNA Ladder. P – Positive Control (*A. baumannii* ATCC BAA–1605). N – Negative Control (*S. aureus* ATCC 29213). **G.** Agarose Gel Electrophoresis of the bla_{OXA-58} amplicons. The expected amplicon band size is 599 bp. Lanes 1–7 (Lanes 1–3 *A. baumannii* AM1–AM3, Lanes 4–7 *A. baumannii* AF1–AF4). Lanes 8–15 (Lanes 8–11 *E. coli* EM1–EM4, Lanes 12–15 *E. coli* EF1–EF4). Lanes 16–23 (Lanes 16–19 *K. pneumoniae* KM1–KM4, Lanes 20–23 *K. pneumoniae* KF1–KF4). Lanes 24–29 (Lanes 24–26 *P. aeruginosa* PM2–PM4, Lanes 27–29 *P. aeruginosa* PF2–PF4). L1 – 1 Kbp DNA Ladder. P – Positive Control (*A. baumannii* TMC 32). N – Negative Control (*S. aureus* ATCC 29213). All putative amplicons of the gene targets were sequenced to confirm gene identities. Only the sequence–confirmed PCR amplicons were used in the analysis. The genes presented include OXA– Oxacillinases.

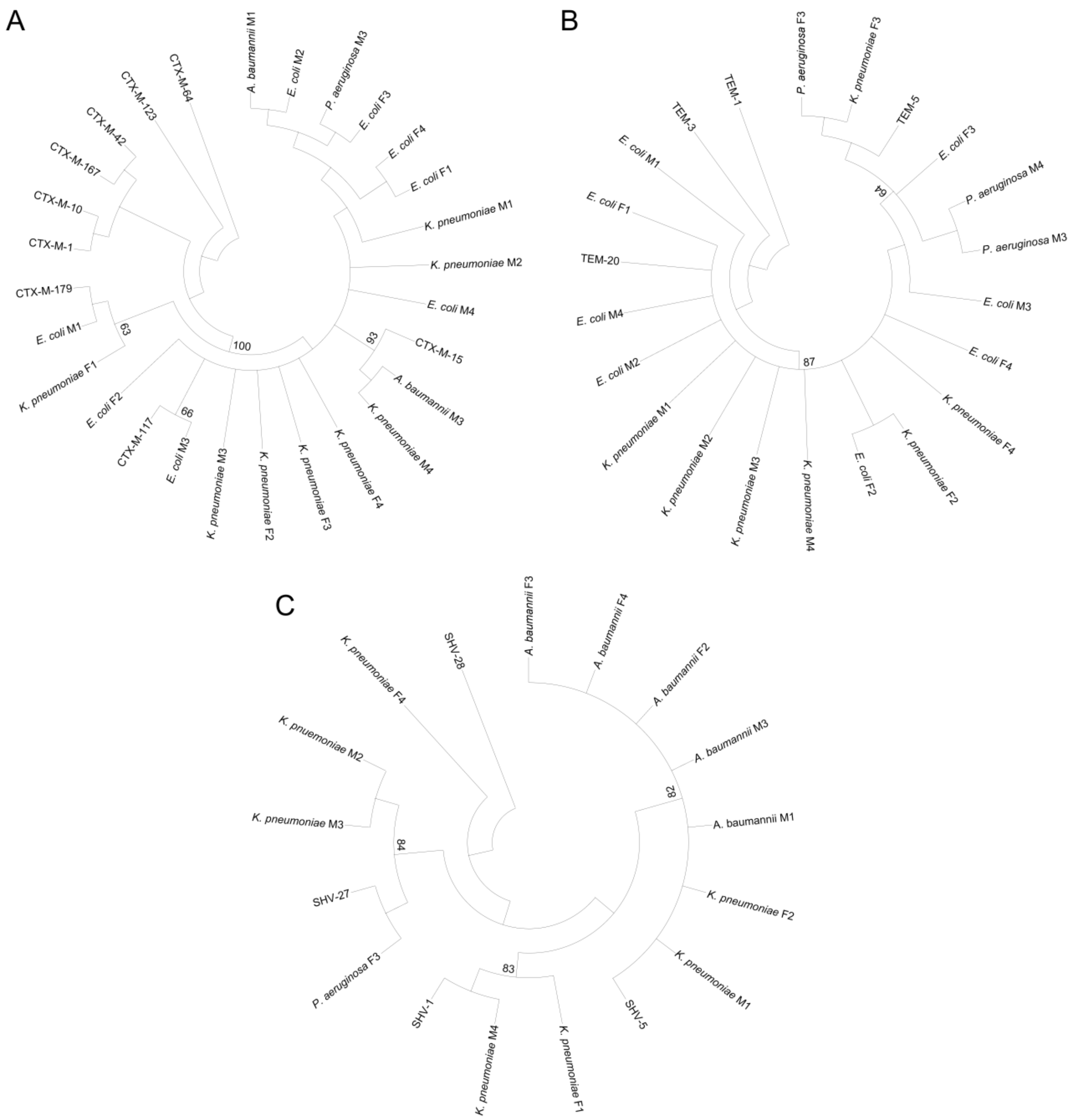

Figure S6. Unrooted maximum likelihood trees of the detected class A β–lactamase genes showed clustering of study isolates' genes in specific gene variants. Panel (A) refers to *bla*$_{\text{CTX–M}}$, (B) to *bla*$_{\text{TEM}}$, and (C) to *bla*$_{\text{SHV}}$ trees. Numbers on the nodes represent bootstrap values out of 1000 replicates. Values less than 50% are not shown. All trees were generated in PhyML version 3.1 using the tree–construction parameters described in Supplementary Table 3. All trees were visualized in TreeExplorer version 2.12. General tree topology and high bootstrap probability values support the relatedness of the isolate's genes to specific reference genes. The genes presented include: CTX–M– Cefotaximase–Munich, TEM– Temoneira β–lactamase, and SHV– Sulfhydryl reagent variable β–lactamase.

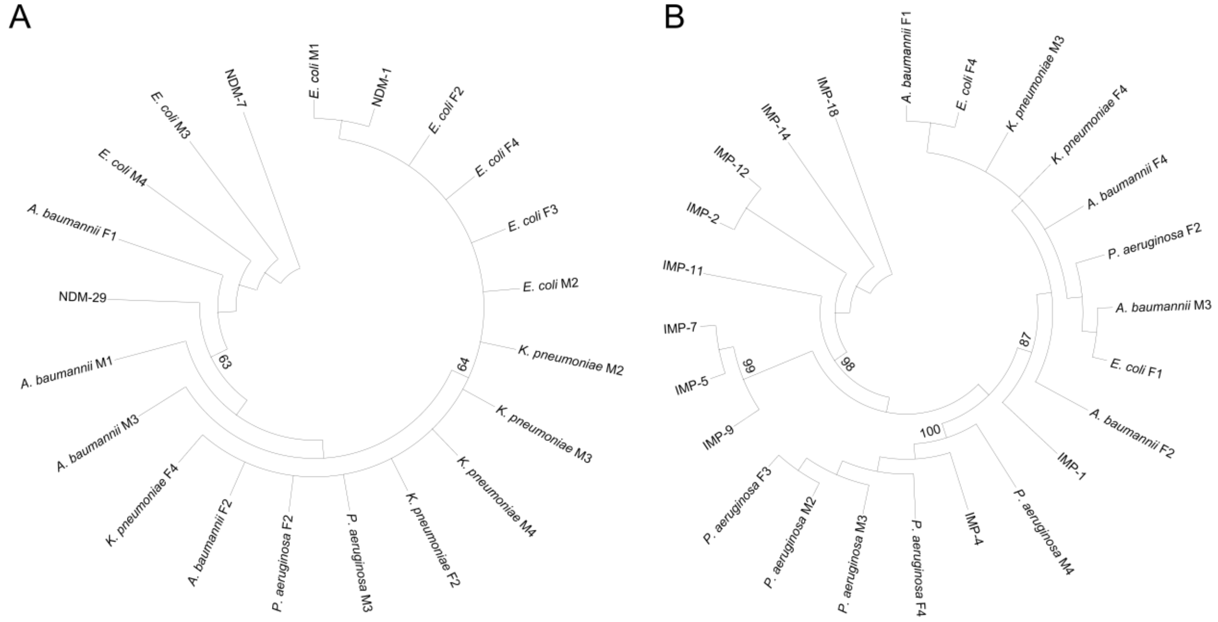

Figure S7. Unrooted maximum likelihood trees of the detected class B β–lactamase genes showed clustering of study isolates' genes in specific gene variants. Panel (A) refers to bla_{NDM}, and (B) to bla_{IMP} trees. Numbers on the nodes represent bootstrap values out of 1000 replicates. Values less than 50% are not shown. All trees were generated in PhyML version 3.1 using the tree–construction parameters described in Supplementary Table 3. All trees were visualized in TreeExplorer version 2.12. General tree topology and high bootstrap probability values support the relatedness of the isolate's genes to specific reference genes. The genes presented include NDM– New Delhi metallo–β–lactamase, and IMP– Imipenemase.

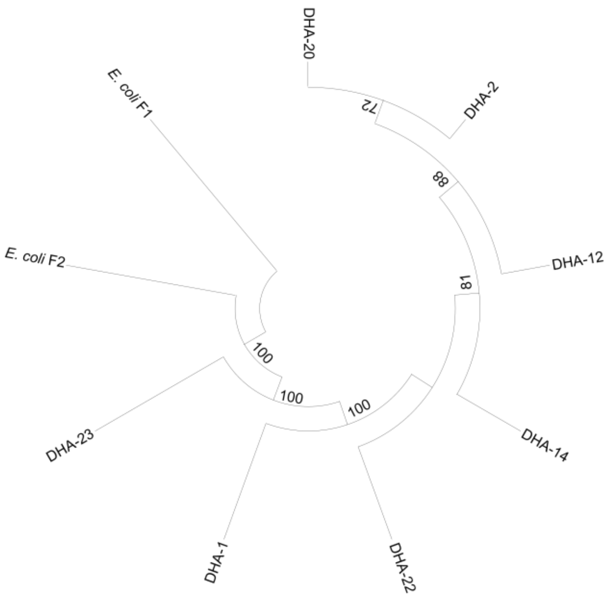

Figure S8. The unrooted maximum likelihood tree of class C *bla*$_{DHA}$ gene showed clustering of study isolates' genes in specific *bla*$_{DHA}$ variants. Numbers on the nodes represent bootstrap values out of 1000 replicates. Values less than 50% are not shown. All trees were generated in PhyML version 3.1 using the tree–construction parameters described in Supplementary Table 3. The tree was visualized in TreeExplorer version 2.12. General tree topology and high bootstrap probability values support the relatedness of the isolate's genes to specific reference genes. The gene presented include DHA– Dharhan β–lactamase.

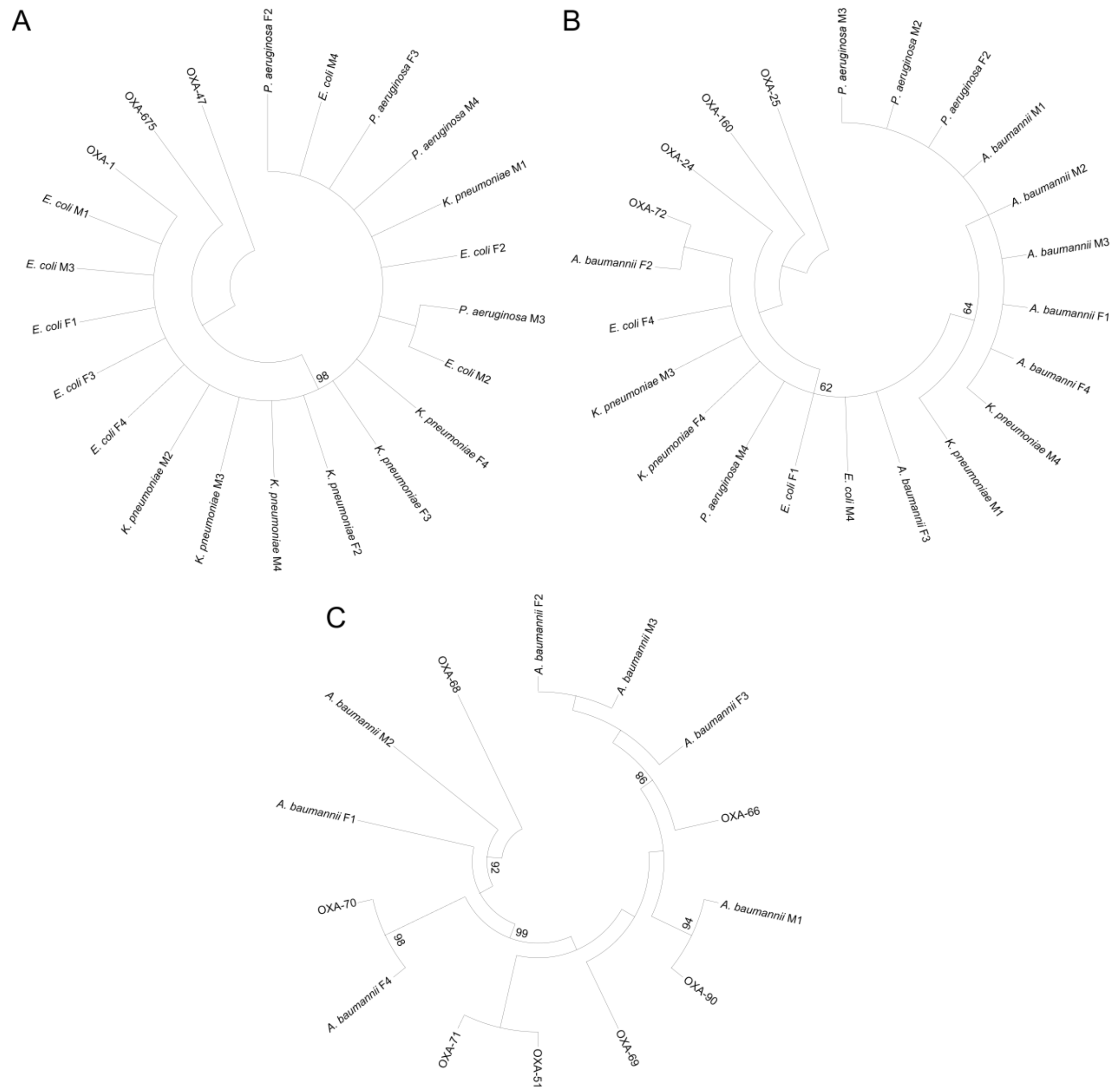

Figure S9. Unrooted maximum likelihood trees of the detected class D β–lactamase genes showed clustering of study isolates' genes in specific gene variants. Panel (A) refers to $\mathit{bla}_{\text{OXA–1}}$, (B) to $\mathit{bla}_{\text{OXA–24–like}}$, and (C) to $\mathit{bla}_{\text{OXA–51–like}}$. Numbers on the nodes represent bootstrap values out of 1000 replicates. Values less than 50% are not shown. All trees were generated in PhyML version 3.1 using the tree–construction parameters described in Supplementary Table 3. All trees were visualized in TreeExplorer version 2.12. General tree topology and high bootstrap probability values support the relatedness of the isolate's genes to specific reference genes. The genes presented include OXA– Oxacillinase.
